# Evaluation of Antibacterial and Anti-oxidant Activities of Suaeda monoica Extract for Its Potential Application

**DOI:** 10.7759/cureus.53091

**Published:** 2024-01-28

**Authors:** Akitha Shakthi, Joseph Linoj, Vasugi Suresh, Mohammed Asif Hussein, Muthamizh Selvamani

**Affiliations:** 1 Physiology, Saveetha Dental College and Hospitals, Saveetha Institute of Medical and Technical Sciences, Saveetha University, Chennai, IND

**Keywords:** innovation, oral pathogens, crude extract, antibacterial activity, antioxidant activity, suaeda monoica

## Abstract

Background: An assessment of *Suaeda monoica* extract's antimicrobial and antioxidant properties was undertaken in light of its possible application as an oral care product. The maintenance of optimal dental health is just as important as overall wellness. Food particles become trapped in the mouth cavity, making it easy for oral bacteria to infect.

Aim: The study sought to ascertain the antibacterial and antioxidant properties of salt marsh *Suaeda monoica *extract.

Materials and methods: Leaves of *Suaeda monoica, *collected, dried and powdered, were dissolved in 70% methanol and the extract of 25-100 μg/ml was analyzed for antioxidant activity through total antioxidant assay, 2,2-diphenyl-1-picrylhydrazyl (DPPH) assay, and total reducing power.* Suaeda monoica* antibacterial activity was also performed and the minimum inhibitory concentration was determined for 75 μg/ml, 100μg/ml, and 150 μg/ml concentrations and tetracycline in 10mcg/disc as a control against three different oral pathogens: *Staphylococcus mutans,* *Streptococcus aureus*, and *Klebsiella spp*.

Results: At varying concentrations of 75 mg/ml to 150 mg/ml, *Suaeda monoica *extracts are efficacious with varying concentrations against the investigated bacterial strains. In the present study, in the DPPH assay, total reducing power, and total antioxidant activity assay, there was an increase in inhibitory percentage as the concentration increased from 25-100 µg/ml, showing maximum inhibition at 100 µg/ml concentration.

Conclusion: The results of the investigation show that* Suaeda monoica *has significant antibacterial and antioxidant activity in a concentration-dependent manner and can be potentially used as an oral care agent after it is assessed for clinical use.

## Introduction

Researchers have spent a lot of time looking into the microorganisms (microbiome) that live in the oral cavity and how they affect human health since the late 20th century. The oral microbiome has been shown to reflect the entire genome of all microorganisms found in the body. Unfortunately, the oral microbiome also contains harmful germs in addition to commensal and symbiotic ones. Further research is required to determine how, among other things, the oral microbiota affects the human body functions [1]. *Streptococcus mutans, Klebsiella sp, and Staphylococcus aureus* (methicillin-resistant *Staphylococcus aureus* (MRSA)) are some of the major oral health-affecting pathogens [2]. The maintenance of optimal oral health is just as important as the maintenance of overall health. Food particles get stuck in the gap between teeth promoting the growth of the microbes. It is crucial to keep the oral cavity in good health because some symptoms may be signs of serious consequences. There are several methods available at the moment for identifying new physiologically active components in medicinal plants [3].

The investigation and explanation of novel antimicrobial, antifungal, and antioxidant components from a variety of natural sources, including, microorganisms, animals, plants, and soil have been the subject of several scientific research endeavors. These commonly accessible traditional herbs could be analyzed and tested in order to find efficacy and benefits with regard to the target microbes. The WHO asserts that cultural acceptance of health, including healthcare practices, health-seeking behavior, and mental well-being, is instrumental in keeping people healthy. For basic oral healthcare, herbal medicine continues to be the mainstay for about 75-80% of the world's population, particularly in industrialized countries, due to its compatibility with the human body and lack of adverse effects [4]. For ages, diverse cultures throughout the world have used herbal medicines to treat a wide range of disorders(cardiovascular, respiratory, and mental disorders). The Siddha system, a traditional Indian system of medicine found by the Siddhars, is one among them. It represented spiritual thinking, detailed observation, and various treatment measures to cure life-threatening conditions like cancer, etc., but all the thoughts of the Siddhars are given as verses scattered in various Siddha literature texts [5].

The development of novel medications from plants has significant potential for commercial therapeutics. Continued research into plant antimicrobials is necessary because, despite their enormous therapeutic potential and success in treating infectious diseases, plant-based antimicrobials still represent a substantial untapped supply of medications [6]. Antimicrobial medications are classified according to the bacteria on which they primarily work [7]. Oxygen is the most crucial ingredient for human survival in our surroundings. Hydroxyl and alkoxy free radicals are extremely reactive species that can cause damage to substances adjacent to cells quickly [8].

Reactive oxygen species (ROS) are known to play a number of in-vivo roles, including phagocytosis, energy production, cell growth regulation, intercellular signaling, and the formation of chemicals having significant physiological consequences [9]. Oxidative stress leads to the activation of the c-Jun N-terminal kinase (JNK)pathway. JNKs are a family of protein kinases that play a crucial role in the regulation of many cellular events, including growth control, transformation, and programmed cell death (apoptosis) [10]. ROS are particularly dangerous because they can destroy proteins, enzymes, and lipids in cellular membranes, resulting in disorders such as Alzheimer's. Parkinson's disease, cancer, atherosclerosis, aging, immunological suppression, and inflammation are all possibilities [11]. An antioxidant reduces free radical generation and storage in tissues, hence preventing tissue depletion [12]. Antioxidants are essential for maintaining oral health. They help to prevent molecules from oxidizing, protecting healthy cells and tissues from oxidative damage. Furthermore, by reducing mouth inflammation, these molecules can reduce the risk of acquiring periodontal diseases. Aside from supplements and topically applied products, antioxidants can be found naturally in certain foods, such as fruits and vegetables. They are essential for avoiding gum disease, tooth decay, and plaque buildup. Antioxidants mediate their protective impact by directly interacting with free radicals, reducing damage to cellular components and averting disease [13].

Antioxidant phytochemicals found in medicinal plants, such as polyphenols, flavonoids, etc. have the ability to act as both antioxidants and radical scavengers. *Suaeda monoica *(*S. monoica*), a type of salt marsh, that belongs to the family *Chenopodiaceae* resembles *Suaeda maritima *in appearance. However, it is a more compact herb with simple, succulent, linear, and juvenile leaves. The twigs are thin and ribbed, and it has edible leaves. Scientific research suggests that *S. monoica* leaf ointment may be used to treat wounds and has antiviral properties due to the presence of triterpenoids and sterols [14]. The current effort aims to characterize the phytochemical makeup and antibacterial efficacy of the leaf extracts from* S. monoica*. The current work is also designed to evaluate the in-vitroantioxidant capabilities of *S. monoica* using a variety of models, including 2,2-diphenyl-1-picrylhydrazyl (DPPH), total antioxidant assay, and total reducing powder.

*S. monoica* is a species of flowering plant in the sea-blite genus *Suaeda*, largely native to the shores of the Indian Ocean from South Africa to Sri Lanka, and salty areas inland. It has been introduced in Argentina. It exhibits phenotypic plasticity, with leaves that are much more succulent when grown under higher salinity conditions. Its leaves are edible, and it is used as an animal fodder plant where it grows. 

The goal of the current investigation was to identify the antibacterial and antioxidant properties of salt marsh *S. monoica *extract and its use as a potential oral care agent.

## Materials and methods

Collection and extraction of samples

**Figure 1:**
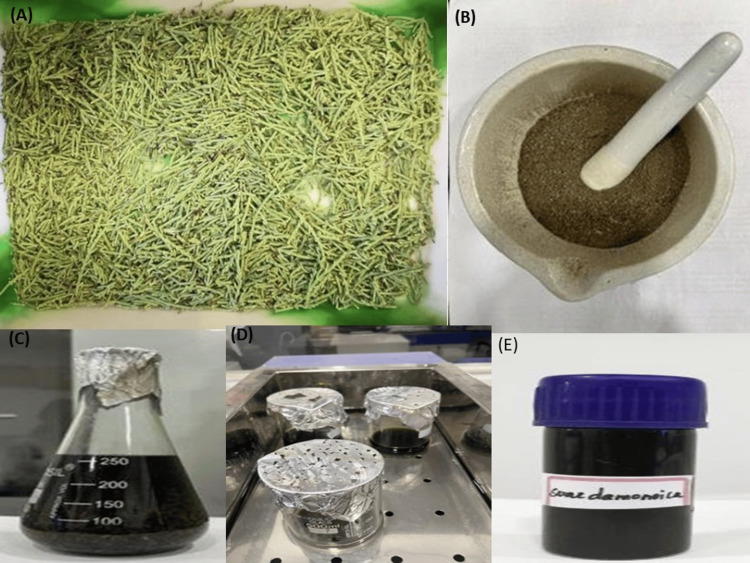
(A), (B) Preparation of the extract. C) Evaporation. D) Aqueous extract. E) Crude extract.

Plant samples of *Suaeda monoica *were collected from Manalmelkudi, Palk Bay, India, and the plant species was identified and authenticated by the Centre of Advanced Study (CAS) in Marine Biology, Annamalai University, Parangipettai, India. The samples were taken to the lab to get rid of the macroscopic epiphytes and other debris, rinsed with distilled water, and then allowed to dry in the shade for two days. *S. monoica* leaves were shade-dried for a week at room temperature (Figure 1A). The dried samples were crushed to fine powder through mortar and pistol and then sieved (Figure 1B). The processed *S. monoica *(100 g) was homogenized with 70% methanol and kept in an orbital shaker for two days at ambient temperature (Figure 1C, 1D). After the evaporation of the solvent, samples were transferred to an airtight container and stored at 4°C (Figure 1E).

Antibacterial assay

The agar disc method was used to examine the secondary metabolites' antibacterial activity. The strains were collected from Saveetha Dental College, Chennai, India. The antibacterial properties were assessed using one Gram-negative bacteria strain (*Klebsiella spp. *(ATCC700834)) and two Gram-positive bacteria strains (*Streptococcus mutans *(ATCC25175) *and Staphylococcus aureus *(ATCC25923). Müeller-Hinton agar and melted nutrients were used to make the medium. The negative control was blank, while the positive control was the common antibiotic tetracycline at a concentration of 10 (μg/disc). The three bacterial strains were tested with 75 (μg/mL), 100 (μg/mL), and 150 (μg/mL) concentrations of secondary metabolites using the agar disc method. The secondary metabolites were dispersed in the Müeller-Hinton agar at concentrations of 75 (μg/mL),100 (μg/mL), and 150 (μg/mL). Each petri plate containing a bacterial lawn was injected with a 1 mL solution of the aforementioned two doses in addition to tetracycline. The diameter of the inhibition zone that formed around the paper disc in each plate was measured after the plates were incubated for 48 hours at 37°C.

MIC (Minimum Inhibitory Concentration) Determination

The minimum inhibitory concentrations against three oral pathogens were tested with different concentrations of the sample. The minimal inhibitory concentrations were determined using the broth microdilution method [15]. A 96-well microtiter plate with a solution of maximal plant extract was prepared [16]. Dimethyl sulfoxide 95:5 was used to prepare serial double dilutions. Each strain's overnight broth culture was made and 50 (µL) of the culture was injected into each well after the final microbe concentration in each well was adjusted to 2x10³ (CFU/ml). For 24 hours, plates were incubated at 37°C. The antibacterial efficiency of the* S. monoica* leaves' extract was investigated [17]. At concentrations of 75 (µg/µl), 100 (µg/µl), and 150 (µg/µl), *S. monoica* was efficacious with varying concentrations against the investigated bacterial strains.

$$\% inhibition = \frac{Control - Sample}{control} * 100$$

In-vitro assay

DPPH Assay

The antioxidant potential of the plant extract was assessed based on its ability to scavenge the stable DPPH free radical. A DPPH solution of the amount of 2900 (µL) was mixed with 100 ml of various crude extract concentrations, and the resulting combination was then dissolved in 120 ml of methanol. After that, the mixture was heated to 37°C and left in the dark for 30 minutes. At 517 nm, the absorbance was determined. The formula given above was used to calculate the percentage of free radical inhibition by DPPH (1%). 

Total Antioxidant Assay

The total antioxidant activity of the green synthesized nanoparticles was determined [18]. In brief, 0.3 ml of samples in various concentrations were made with 3 ml of reagent solution (0.6 M sulphuric acid, 4 M ammonium molybdate). In a water bath, the reaction mixture was incubated for 90 minutes at 95°C. At 695 nm, the absorbance of all sample mixes was measured. As per the given formula, total antioxidant activity has been reported as a percentage.

Total Reducing Powder

The total reducing power was evaluated at various concentrations ranging from 25-100 (μg/ml). In detail, 2.5 ml of benzene chloroform and 2.5 ml of a reaction mixture containing potassium ferrocyanide (1%), together with 1 ml of benzene chloroform (2:1) comprising varying concentrations of crude extract were combined. After that, the mixture was incubated for 20 minutes at 50°C. After the incubation period, 2.5 ml of 10% trichloroacetic acid was added, and 2.5 ml was centrifuged at 10,000 rpm for 10 minutes. The absorbance at 700 nm was measured after adding 2.5 ml of distilled water and 0.5 ml of ferric chloride (FeCl_3_) (0.1%) to the top layer. As a positive control, ascorbic acid was used. 

## Results

Antibacterial activity

**Figure 2:**
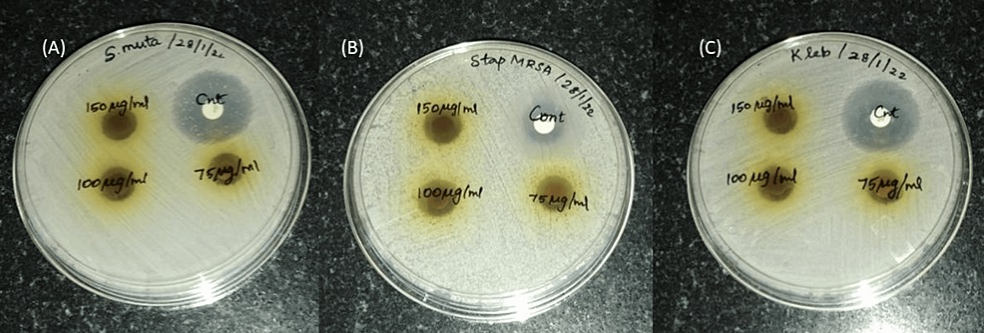
Determination of MIC for three oral pathogens, A) 75 μg/ml, B) 100 μg/ml, and C) 150 μg/ml. MIC: minimum inhibitory concentration

The zone of inhibition for *Streptococcus mutans *compared with the standard was found as 9, 9.5, and 9.5 mm at the concentrations of 75, 100, and 150 (µg/ml), respectively. The zone of inhibition for *Klebsiella s*pp. was found as 8, 8, and 9 mm at concentrations of 75, 100, and 150 (µg/ml). The zone of inhibition for *Staphylococcus aureus* was noted as 8.5, 9.5, and 10 mm at concentrations of 75, 100, and 150 (µg/ml). As per our present investigation, the zone of inhibition was examined and compared with the control tetracycline as shown in Figure 2A-2C. The three bacterial strains were assessed with tetracycline as positive control. Against *Streptococcus mutans, *it showed a zone of inhibition of 19 mm, against *Staphylococcus aureus *of 10 mm, and against *Klebsiella spp.* of 19 mm. The extract affected all the bacteria strains, *Staphylococcus mutans, Streptococcus aureus, and Klebsiella spp. *

Antioxidant assays

**Figure 3:**
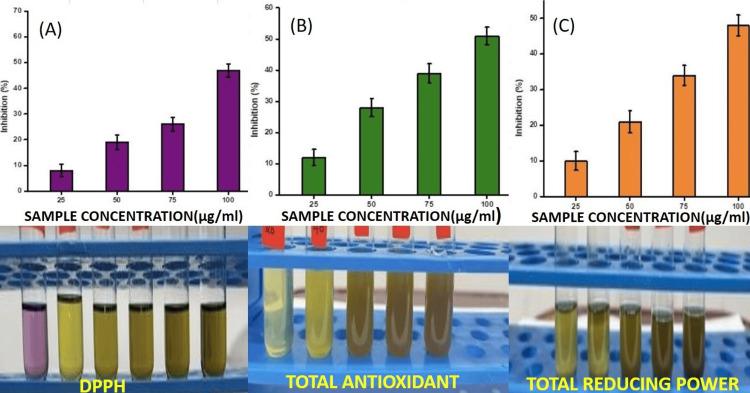
(A) DPPH assay. (B) Total antioxidant. (C) Total reducing power. DPPH: 2,2-diphenyl-1-picrylhydrazyl

Superoxide radicals are a known precursor to more reactive species and are incredibly destructive to biological components. The results show unequivocally that *S. monoica* leaf extracts have detectable superoxide radical scavenger properties. In the present study, in the DPPH assay, total reducing power, and total antioxidant activity assay, there was an increase in the percentage of inhibition as the concentration increased from 25-100 (µg/ml) showing maximum inhibition at 100 (µg/ml) concentration. In the DPPH assay, at a concentration of 75 (µg/ml), there was 30% inhibition (Figure 3A). In the total reducing power at 75 (µg/ml), there was 40% inhibition (Figure 3B). In the total antioxidant assay at 75 (µg/ml), 45% of inhibition was observed (Figure 3C). The study found a positive correlation between the DPPH, total reducing power, and total antioxidant assay of the extract, with the highest correlation coefficient (r=0.95). There is a 95% chance that the same rationale, mechanism, or bioactive components are responsible for the extract's antioxidant activity. As a result, a more focused separation method that focuses on the plant's bioactive components may be supported by their chemical composition and biological activities, which could improve medication development. *S. monoica* extracts have been shown to have potential antibacterial activity against *Streptococcus mutans*, *Klebsiella spp.*, and *Staphylococcus aureus* (MRSA), which are some of the major oral-health-affecting pathogens. Thus, the antioxidant and antibacterial qualities of *S. monoica* determined in the present study may find possible applications in oral care products, which may be considered in the development of eco-friendly and effective dental care products.

## Discussion

It is becoming more important to focus on traditional herbal medicinal plants from effective scientific viewpoints as chronic illnesses increasingly become the leading causes of morbidity and death globally. It is desirable to cover as many targets as possible concurrently with as many active principles in a unique and balanced manner [19]. It is also essential to note that the Indian subcontinent is well known for its diverse culture and plant life, and many people still rely on traditional medicinal herbs to treat a variety of illnesses. It is essential to document this unique information relating to the ethnic population [20] before this culture is lost with the loss of biodiversity. It is also important to conduct organized research to reestablish and validate the therapeutic potential of various medicinal plants that are not systematically documented in the literature.

The antibacterial activity of *S. monoica *is a sign that bioactive secondary metabolites were produced. *S.monoica* has a variety of bioactive substances with vast possibilities of antibacterial action against a range of diseases because they function without any negative side effects. These findings support the possibility of using the chosen plant extract as a bioactive agent with antibacterial activity [21]. *S. monoica* has potent antibacterial activity against the three oral pathogenic bacterial strains, *Staphylococcus mutans, Streptococcus aureus, and Klebsiella spp.,* that were included in the study. As per our present investigation, the zone of inhibition was examined and compared with the control Tetracycline as shown in Figure 2A-2C. The extract affected all the bacteria. *Staphylococcus mutans*, however, demonstrated the most antibacterial action. 

Many human illnesses are considered to be significantly influenced by free radicals and other reactive entities. Because free radicals have a harmful function in biological systems, radical scavenging activities are crucial. In their study, Watanabe et al [22] showed the presence of several secondary metabolites, such as flavonoids, and these compounds often contribute to the antioxidant properties of the plant [22]. Other compounds, such as phenolic compounds, have antioxidant and scavenging capabilities. The investigation's findings unequivocally demonstrate the potent antioxidant qualities of the *S. monoica* leaf extract. This study examined the ability of *S. monoica* leaf extract to reduce and scavenge superoxide hydroxyl, DPPH, and 2,2′-azino-bis-(3-ethylbenzothiazoline-6-sulfonic) acid (ABTS) radical cations in petroleum ether, benzene, ethyl acetate, methanol, and ethanol in order to determine their in-vivo antioxidant activity. Using Trolox and ascorbic acid as reference standard antioxidants, these techniques have shown how effective the extracts are.

An investigation was conducted on the in-vitro antibacterial efficacy of ethanol extracts from eight plant species that are traditionally used in South Africa to treat oral health issues. Generally speaking, when the minimum inhibitory concentration (MIC) and minimum bactericidal concentration (MBC) of ethanol extracts against these microorganisms were determined by microdilution, Gram-negative bacteria were found to be more resistant to the plant extracts than Gram-positive bacteria [23]. Antioxidants are endogenous or exogenous substances that protect the biological system from the harmful effects of oxidative stress in any form [24]. An antioxidant prevents the formation of free radicals and their storage in tissues, thus helping to prevent tissue depletion [25]. Within the intricate signaling network of cells, ROS play a significant regulatory role. They are essential for immune response, adaptation to metabolic and physiological stresses, cell growth and differentiation, and defense against pathogen invasion. However, a number of factors can disrupt normal cellular functions and expose tissues to oxidative stress conditions, which can lead to an overabundance of ROS [26]. In the present study, methanol extracts from *S. monoica* leaf exhibited substantial antioxidant activity, according to the findings from DPPH free radical scavenging methods. In every test technique, it was discovered that the extracts had varying degrees of antioxidant activity and antibacterial activity. A notable antioxidant activity and antibacterial activity against human pathogens was discovered, according to this investigation. The high concentration of phenols and flavonoids present in the leaf extract indicates that these chemicals may have a significant role in the antioxidant and antibacterial activity.

Limitations

To make progress in this field of research, it will be crucial to overcome restrictions by employing rigorous experimental design, conducting extensive characterization, and giving serious thought to prospective applications and safety considerations. Moreover, engaging in collaborative efforts with specialists in the fields of microbiology, biotechnology, and pharmacology can help overcome some obstacles. Additionally, It is vital to acknowledge that the current investigation focused on in-vitro assessments. In order to enhance the credibility of the *S. monoica* leaf extracts, it is essential to conduct in-vivo research and clinical trials for the purpose of validating its effectiveness and safety. Additionally, the chemical structure of the drug can also be viewed and analyzed. 

## Conclusions

*S. monoica* possesses antioxidant and antibacterial qualities that may find possible application in dental care products; this inspired the creation of the current efficient and environmentally friendly process. *S. monoica *extracts have been shown to have antibacterial activity against *Streptococcus mutans, Klebsiella spp*., and S*taphylococcus aureus* (MRSA). This has raised speculation regarding the potential role of the extract in controlling the development of antibacterial resistance, while its antioxidant properties suggest a potential role in preventing oxidative damage to oral tissues. When *S.monoica* is evaluated for clinical use, the investigation's findings also support the possibility of its use as an oral care agent.
